# Time to publication of oncology trials and why some trials are never published

**DOI:** 10.1371/journal.pone.0184025

**Published:** 2017-09-21

**Authors:** Paul B. Chapman, Nathan J. Liu, Qin Zhou, Alexia Iasonos, Sara Hanley, George J. Bosl, David R. Spriggs

**Affiliations:** 1 Department of Medicine, Memorial Sloan Kettering Cancer Center, New York, New York, United States of America; 2 Weill Cornell Medical College, New York, New York, United States of America; 3 Epidemiology-Biostatistics, Memorial Sloan Kettering Cancer Center, New York, New York, United States of America; Universitatsspital Basel, SWITZERLAND

## Abstract

**Background:**

Very little is known about the proportion of oncology trials that get published, the time it takes to publish them, or the reasons why oncology trials do not get published.

**Methods:**

We analyzed all clinical trials that closed to accrual at our cancer center between 2009–2013. Trials were categorized by study purpose (therapeutic vs. diagnostic), phase (pilot, phase I, II, or III), and sponsor (industrial, cooperative group, institutional, or peer-reviewed). Final publications were identified in MEDLINE and EMBASE by NCT numbers, or by querying the principal investigator. For trials not published, we surveyed the principal investigators to identify the reason for non-publication.

**Findings:**

469 of 809 protocols (58%) had been published by November 2016. The calculated probability of publication 7 years after completing accrual was 70.4%; the calculated median time to publication was 47 months. Only 18.8% of protocols overall were estimated to be published within 2 years from completing accrual. The calculated probability of publication was higher for therapeutic trials than non-therapeutic trials, but there was no difference based on phase or sponsor. Among protocols not published, 45.3% had completed accrual, and among these, a majority had a manuscript in preparation or review, or the trial was still collecting data. Failure to publish due to a pharmaceutical sponsor was rare. 30.6% of unpublished trials had closed for various reasons before completing accrual, usually due to poor accrual or pharmaceutical sponsor issues.

**Interpretation:**

Almost 30% of trials were calculated to be unpublished by 7 years after closing to accrual at our institution. Failure to reach accrual goals was an important factor in non-publication. We have devised new institutional policies that identify trials likely not to meet accrual goals and require early closure. We should be able to shorten the time from accrual completion to publication, especially for pilot and phase I trials for which long follow up is not needed.

## Introduction

It is axiomatic among the research and patient communities that medical progress is dependent on clinical trials. As a result, both communities and the regulatory authorities have been concerned with optimizing access of patients to clinical trials and experimental new treatments, streamlining clinical trial design, and shortening the time to new drug approvals. Relatively little attention has been paid to efficiency of the clinical trial publication process.

Many observers have reported that positive clinical trials are more likely to be published than negative trials [1–7]. Other factors also affect the odds of publication such as whether or not the trial was industry-sponsored [8–11], was randomized [10], or was considered a pivotal trial [12]. Overall, the publication rates of clinical trials have generally been reported between 60–70% (range 46–86%)[8, 10, 12–15]. However, these analyses included clinical trials from a wide range of medical fields; oncology trials represented only a minority in most of these analyses and the reasons for non-publication were not explored.

Among oncology trials specifically, little has been published about the overall rate of publication or reasons for trials not to be published. Chen reported on 598 randomized oncology trials world-wide and found a 66.6% cumulative probability of publication with only 32% of the trials having been published within 24 months of the trial completion date [4]. They found that publication was less likely in trials with blinded assignment to treatment groups and in trials considered to have a negative outcome.

We examined clinical trials that closed to accrual between 2009–2013 at our cancer center to explore how long it took to publish the results, how many had not been published, and the reasons for non-publication.

## Methods

### Data collection

Using an institutional database, we identified all trials at Memorial Sloan Kettering Cancer Center (MSKCC) with a date of accrual closure between Jan 1, 2009 and Dec. 31, 2013. All trials had been approved by the MSKCC Institutional Review Board. These dates were chosen to ensure a minimum of three years of follow-up. November 1, 2016 was the date of last follow-up for publication status. All trials opened to accrual at MSKCC were included in this analysis except for studies not intended for independent publication such as specimen collection or expanded access trials. From our institutional database, we obtained start and stop dates of study accrual at MSKCC, number of patients accrued, study protocols, ClinicalTrials.gov registry (NCT) numbers, study purpose (therapeutic vs. diagnostic), and study phase (pilot, phase I, II, or III). We also categorized the studies based on the study sponsor according to the NCI Cancer Center Support Grant guidelines based on funding and who controlled the design and implementation of the study: Industrial (pharmaceutical company), Institutional (MSKCC or another academic institution), national cooperative group, or peer-reviewed (funded by other organizations employing external peer review). This was done in order to analyze outcomes by study sponsor type.

The primary endpoint of our study was to estimate the proportion of clinical trials that were ultimately published. Secondary endpoints were time to publication with special attention to 2-yr publication rates and median time to publication. The 2-yr publication rate was selected as a measure of timely publication as noted by previous investigators [4, 13] Among unpublished trials, we collected data on the reason for non-publication. Time to publication was computed as the time elapsed between the date of accrual closure at MSKCC and the date of publication. We selected date of accrual closure rather than date of study closure as our time zero because final study closure can often occur many years after accrual has been completed and after an initial publication has appeared. We defined a publication as a research article communicating the primary outcomes of a trial in any peer-reviewed journal. Publications linked to NCT numbers in the MEDLINE and EMBASE databases were retrieved for 235 trials. For the remainder (574 studies without an easily-identifiable publication), a survey was sent to the principal investigator (PI) at MSKCC of each trial to retrieve peer-reviewed publications of trial results. All publications were independently reviewed by one of the authors (NJL) to ensure agreement between the published result and the primary aims of the trial as delineated in the study protocol. When available, the time of first publication online was recorded as the date of publication. If not provided, the issue date of the article was recorded. The day of publication within the month was assumed to be the 15^th^ if no specific day of the month was given.

If the trial was not published, the PI was asked to select one main reason for non-publication among several predetermined options (Table 1).

**Table 1:** Response options in reporting reasons for non-publication of trials.

Categories	Response options
TRIAL COMPLETED ACCRUAL: DATA COLLECTION COMPLETE	Completed-manuscript in preparation
	Completed-data not interesting
	Completed-manuscript in review or rejected
	Completed-sponsor delaying publication
	Completed-no time to write
TRIAL COMPLETED ACCRUAL: DATA COLLECTION IN PROGRESS	Incomplete-data analysis ongoing
TRIAL DISCONTINUED BEFORE COMPLETING ACCRUAL	Discontinued-due to poor accrual
	Discontinued-by sponsor for other reason
	Lost to follow-up-PI left MSK
	Discontinued-due to toxicity
	Discontinued-due to drug availability
OTHER	Trial never opened at MSK
	Reason Unknown

### Statistical analysis

Protocols with a published research article communicating the primary outcomes of a trial in any peer-reviewed journal were considered events. For 340 protocols without a publication, 278 were censored on 8/01/2016 and 62 were censored on 10/01/2016 depending on the date the PI was last contacted. Kaplan-Meier method was used to estimate the probability of publication $Prob(publictaion<t)=1-\hat{S}(t)$, where $\hat{S}(t)$ is the Kaplan Meier estimate. The probability of publication at 2 years was reported and compared through Z-test (for binary covariates) or Chi-squared test (for categorical covariates)[16]. The log-rank test was also reported for overall differences in the distribution of the cumulative probability of publication among different types of trials.

Conditional probability[16] was estimated as:

$$Prob\{t_{i}<publication<t_{j}|publication\geq t_{i}\}=1-\frac{Prob\{publication\geq t_{j}\}}{Prob\{publication\geq t_{i}\}}=1-\frac{\hat{S(t_{j})}}{\hat{S(t_{i})}}$$

The variance of this probability was calculated based on “Greenwood” formula[17] as ${\left[\frac{Prob\{publication\geq t_{j}}{Prob\{publicaiton\geq t_{i}}\right]}^{2}\sum_{k=i+1}^{j}\frac{d_{k}}{r_{k}(r_{k}-d_{k})}$. The 95%CI was constructed assuming normal distribution. All analyses were performed in SAS 9.4 or R 3.2.3.

Descriptive statistics were reported for non-publication reasons. Fisher’s exact test was used to compare the non-publication reasons between different trial categories.

## Results

### Protocols analyzed

**Fig 1:**
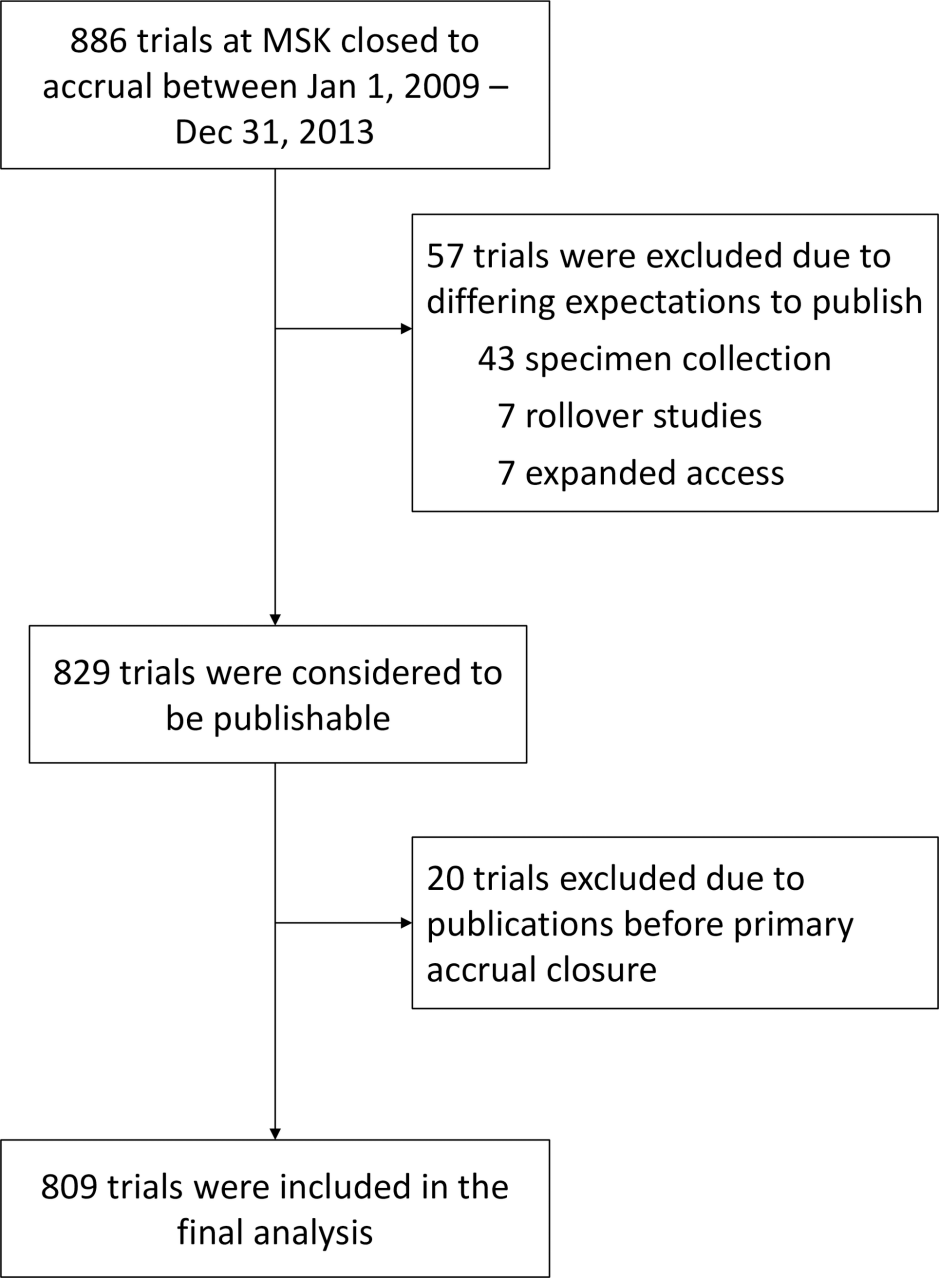
Consort diagram.

**Fig 2:**
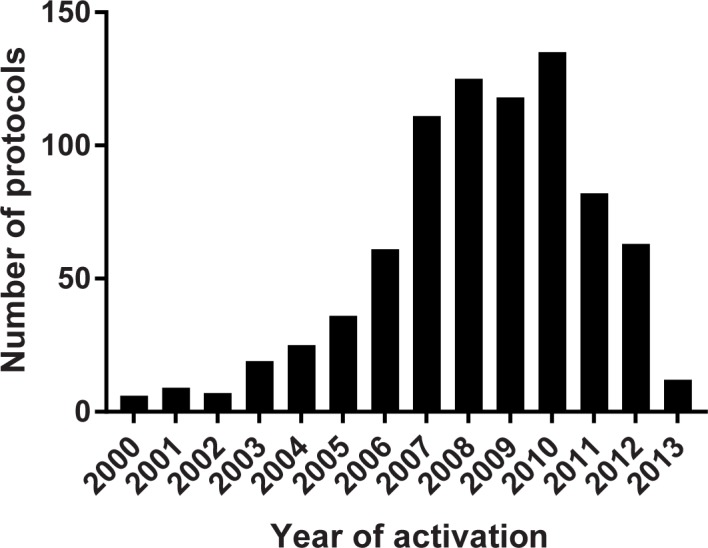
Distribution of activation years. The year of activation at MSKCC is shown on the *x*-axis for the 809 protocols analyzed. Protocols for the year 2000 (N = 6 protocols) include 3 protocols activated in earlier years (1977, 1998, 1999). These protocols were collapsed into 2000 for clarity of the histogram.

Between 2009–2013, 886 trials closed to accrual at MSKCC. Fifty-seven trials designed primarily for specimen collection, treatment continuation, or expanded access were excluded from this analysis because they were not designed for independent publication. Another 20 trials were excluded because they had been published prior to the date of closure to accrual. This left 809 trials that formed the basis of this analysis (Table 2, Fig 1). The year of activation ranged from 1977 to 2013 (Fig 2). The median year was 2009 (25%-75%; 2007–2010). Six hundred twelve trials (76%) had therapeutic endpoints; 197 (24%) were non-therapeutic trials. Trials included 109 pilot studies (13%), 181 phase I trials (22%), 290 phase II trials (36%), and 121 phase III trials (15%). The remaining 108 trials (13%) did not fall into these categories and were considered “other”. Sponsorship was divided between 286 industrially-sponsored trials (35%), 119 national cooperative group studies (15%), 346 trials institutionally sponsored (43%), and 58 trials externally peer-reviewed grants (7%).

**Table 2:** Protocol characteristics, overall observed, and estimated 2-year publication rates.

	Total	%^§^	Published	% published at time of analysis	Estimated 2-Yr publication rate (95%CI)	p-value*
All protocols	809	100	469	56	18.8% (16.3–21.7%)	
Purpose						
Therapeutic	612	76	367	45	18.8% (15.9–22.1%)	0.998
Non-therapeutic	197	24	102	12.5	18.8% (14–25%)	
Phase						
Pilot	109	13.5	53	6.5	18.3% (12.3–27%)	0.128
I	181	22	101	12.5	20.4% (15.3–27.1%)	
II	290	36	175	21.5	19.7% (15.5–24.7%)	
III	121	15	77	9.5	11.6% (7–18.8%)	
Other	108	13.5	63	8	22.2% (15.5–31.3%)	
Sponsor						
Industrial	286	35	174	21.5	18.5% (14.5–23.5%)	<0.001
National cooperative group	119	15	66	8	5.9% (2.8–11.9%)	
Institutional	346	43	197	24	22% (18–26.7%)	
Peer-reviewed	58	7	32	4	27.6% (17.9–41%)	

*Test for the difference of cumulative probability at 2 years.

^§^Percentages may not add up to 100 due to rounding.

Of the 574 trials for which we queried the PI to identify unindexed publications, we found publications for 234 and confirmed that 337 had not been published. For 3 trials, we could not get any information and counted them as having not been published, reasons unknown. Overall, 469 (58%) of the trials had been published by the data cutoff date.

### Cumulative probability of publication

**Fig 3:**
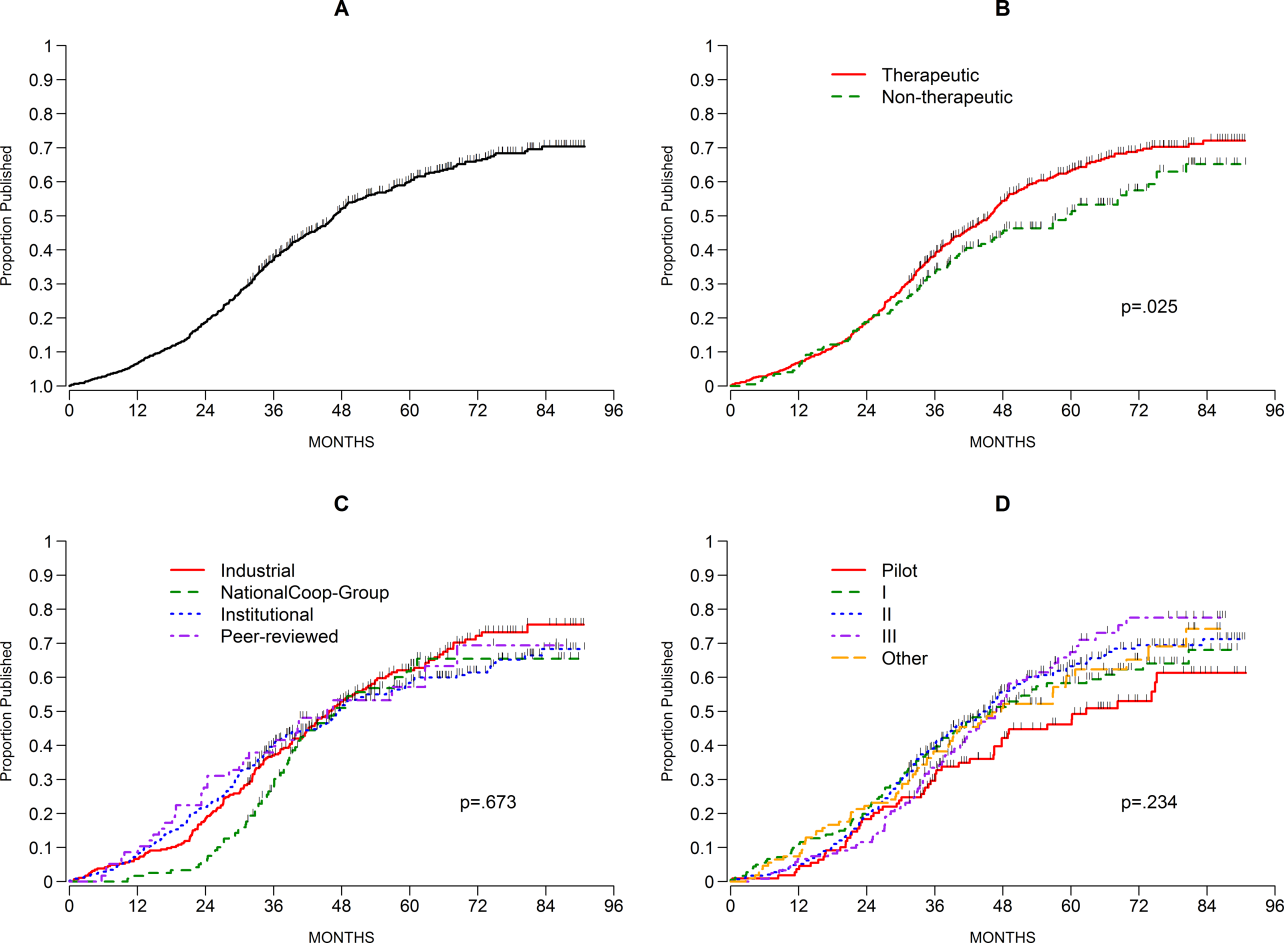
Cumulative probability of publication. A) All protocols. Tick marks indicated censored (non-published) trials. B) Therapeutic (red) and non-therapeutic (green) protocols were plotted separately. Log-rank p = 0.025. C) Cumulative probability of publication based on type of sponsor. There was no significant differences between trials sponsored by industry (red line), National cooperative group trials (green), institutional sponsors (broken blue line), or by peer-reviewed grants (broken purple line). Log rank p = 0.673. D) Cumulative probability of publication based on phase of the trial. There was no significant differences between pilot trials (red), phase I (broken green line), phase II (broken blue line), phase III (broken purple line), and other trials (yellow) were plotted separately. Log rank p = .234.

At the time of data lock, 469 of the 809 protocols (58%) had been published. We measured from the time of completion of accrual at MSKCC. For all protocols, the estimated probability of publication 7 years after closure to accrual was 70.4% (Fig 3A). The estimated median time to publication was 47 months after closure to accrual.

Fig 3B shows that the cumulative probability of publication over the entire time period was significantly different for therapeutic compared to non-therapeutic trials (log-rank p = 0.025). Although the difference in the proportion of trials published by 2 years was negligible, therapeutic trials had a higher cumulative publication probability by 7 years (72.1%; 95%CI 67–77) compared to non-therapeutic trials (65.2%; 95%CI 55.5–74.7). One possibility is that the non-therapeutic trials became less relevant after several years and were not considered of interest (discussed below). There were no significant differences in the cumulative probability of publication by sponsor type (Fig 3C). National cooperative group trials had a very low publication proportion by year 2 (5.9%; 95%CI 2.8–11.9%), presumably because many of these trials require longer periods of time to collect data and follow up. By year 4, the proportion of protocols published had caught up with the other trials.

Surprisingly, there was also no difference by trial phase (pilot, phase I, II, III, or other) (Fig 3D). Given that pilot and phase I trials generally require little follow up after patients complete treatment, we had expected to see a shorter time to publication for these trials.

### 2-year publication rate

Overall, 18.8% were estimated to have been published within 2 years of closure to accrual (Table 2). We observed no significant difference in publication by 2 years according to purpose of the trial (therapeutic vs. non-therapeutic) or on the phase of the trial. However, national cooperative group trials had a lower publication proportion at 2 years (5.9%) compared to trials that were industry-sponsored (18.5%), institution sponsored (22%), or peer-reviewed (27.6%).

### Conditional probabilities of publication

For trials not published by 2 years, we calculated the conditional probability for publication by year 3 and by year 7 (Table 3). The probability these trials were published by the next year (year 3) was 22% overall; the probability of these trials were published by year 7 was 64%, overall. Therefore, there was a 36% chance that trials not published by year 2 would remain unpublished by year 7. There were no obvious differences in the conditional probability of publication based on trial purpose, phase, or sponsor, although formal statistical analyses were not applied.

**Table 3:** Conditional probabilities of publication for trials not published by year 2.

	Probability of publication at year 3	Probability of publication by year 7
All	0.22(0.19–0.26)	0.64(0.58–0.69)
Purpose		
Therapeutic	0.24(0.2–0.28)	0.66(0.6–0.72)
Non-therapeutic	0.18(0.12–0.24)	0.57(0.46–0.69)
Phase		
Pilot	0.14(0.07–0.21)	0.53(0.38–0.67)
I	0.23(0.16–0.3)	0.6(0.47–0.73)
II	0.25(0.19–0.3)	0.64(0.56–0.73)
III	0.25(0.16–0.33)	0.75(0.63–0.86)
Other	0.21(0.12–0.29)	0.67(0.5–0.84)
Sponsor		
Industrial	0.23(0.17–0.28)	0.7(0.61–0.79)
National cooperative group	0.22(0.14–0.3)	0.63(0.52–0.74)
Institutional	0.23(0.18–0.28)	0.59(0.5–0.68)
Peer-reviewed	0.17(0.05–0.28)	0.58(0.34–0.81)

### Reasons for protocols not being published

We collected information from the principal investigators at MSKCC as to the reason why the unpublished trials had not been published. Among the 340 protocols that had not been published, 154 (45.3%) completed data collection and analyses. For 93 (60%) of these completed but unpublished trials, a manuscript was either in preparation or had been submitted for publication (Table 4). Of the remaining unpublished completed trials, 45 (29%) were not considered of sufficient interest by the investigators. Many of these were either “negative trials” or were no longer considered of interest to the field. Of note, there were only 11 completed trials that had not been published due to sponsor delays (3.2% of all unpublished trials; 1% of all trials). Sixty trials (17.6% of unpublished trials; 7% of all trials) were unpublished due to ongoing data collection and/or analyses accounted for. Overall survival is a common endpoint in oncology trials and may explain some of this delayed publication.

**Table 4:** Reasons trials were not published.

	ALL	PURPOSE		PHASE					SPONSOR			
		Therapeutic	Non-therapeutic	Industrial	National Coop. Group	Insti- tutional	Peer-reviewed	Other	Industrial	National Coop. Group	Instit.	Peer-reviewed
**TRIAL COMPLETED ACCRUAL**	**154(45.3%)**	**109(44.5%)**	**45(47.4%)**	**46(41.1%)**	**15(28.3%)**	**75(50.3%)**	**18(69.2%)**	**23(51.1%)**	**46(41.1%)**	**15(28.3%)**	**75(50.3%)**	**18(69.2%)**
Completed-manuscript in preparation	68(20%)	53(21.6%)	15(15.8%)	18(16.1%)	10(18.9%)	31(20.8%)	9(34.6%)	8(17.8%)	18(16.1%)	10(18.9%)	31(20.8%)	9(34.6%)
Completed-data not interesting	45(13.2%)	27(11%)	18(18.9%)	10(8.9%)	2(3.8%)	27(18.1%)	6(23.1%)	7(15.6%)	10(8.9%)	2(3.8%)	27(18.1%)	6(23.1%)
Completed-manuscript in review or rejected	25(7.4%)	17(6.9%)	8(8.4%)	7(6.3%)	3(5.7%)	14(9.4%)	1(3.8%)	4(8.9%)	7(6.3%)	3(5.7%)	14(9.4%)	1(3.8%)
Completed-sponsor delaying publication	11(3.2%)	10(4.1%)	1(1.1%)	10(8.9%)	0(0%)	1(0.7%)	0(0%)	2(4.4%)	10(8.9%)	0(0%)	1(0.7%)	0(0%)
Completed-no time to write	5(1.5%)	2(0.8%)	3(3.2%)	1(0.9%)	0(0%)	2(1.3%)	2(7.7%)	2(4.4%)	1(0.9%)	0(0%)	2(1.3%)	2(7.7%)
**TRIAL IN PROGRESS**	**60(17.6%)**	**46(18.8%)**	**14(14.7%)**	**15(13.4%)**	**25(47.2%)**	**19(12.8%)**	**1(3.8%)**	**6(13.3%)**	**15(13.4%)**	**25(47.2%)**	**19(12.8%)**	**1(3.8%)**
Incomplete-data analysis ongoing	60(17.6%)	46(18.8%)	14(14.7%)	15(13.4%)	25(47.2%)	19(12.8%)	1(3.8%)	6(13.3%)	15(13.4%)	25(47.2%)	19(12.8%)	1(3.8%)
**TRIAL DISCONTINUED BEFORE COMPLETING ACCRUAL**	**104(30.6%)**	**76(31%)**	**28(29.5%)**	**44(39.3%)**	**11(20.8%)**	**44(29.5%)**	**5(19.2%)**	**11(24.4%)**	**44(39.3%)**	**11(20.8%)**	**44(29.5%)**	**5(19.2%)**
Discontinued-due to poor accrual	45(13.2%)	29(11.8%)	16(16.8%)	8(7.1%)	8(15.1%)	25(16.8%)	4(15.4%)	8(17.8%)	8(7.1%)	8(15.1%)	25(16.8%)	4(15.4%)
Discontinued-by sponsor for other reason	35(10.3%)	29(11.8%)	6(6.3%)	24(21.4%)	2(3.8%)	9(6%)	0(0%)	2(4.4%)	24(21.4%)	2(3.8%)	9(6%)	0(0%)
Lost to follow-up-PI left MSK	13(3.8%)	8(3.3%)	5(5.3%)	4(3.6%)	0(0%)	8(5.4%)	1(3.8%)	1(2.2%)	4(3.6%)	0(0%)	8(5.4%)	1(3.8%)
Discontinued-due to toxicity	7(2.1%)	7(2.9%)	0(0%)	6(5.4%)	0(0%)	1(0.7%)	0(0%)	0(0%)	6(5.4%)	0(0%)	1(0.7%)	0(0%)
Discontinued-due to drug availability	4(1.2%)	3(1.2%)	1(1.1%)	2(1.8%)	1(1.9%)	1(0.7%)	0(0%)	0(0%)	2(1.8%)	1(1.9%)	1(0.7%)	0(0%)
**OTHER**	**22(6.5%)**	**14(5.7%)**	**8(8.4%)**	**7(6.3%)**	**2(3.8%)**	**11(7.4%)**	**2(7.7%)**	**5(11.1%)**	**7(6.3%)**	**2(3.8%)**	**11(7.4%)**	**2(7.7%)**
Trial never opened at MSK	19(5.6%)	11(4.5%)	8(8.4%)	6(5.4%)	1(1.9%)	10(6.7%)	2(7.7%)	5(11.1%)	6(5.4%)	1(1.9%)	10(6.7%)	2(7.7%)
Reason Unknown	3(0.9%)	3(1.2%)	0(0%)	1(0.9%)	1(1.9%)	1(0.7%)	0(0%)	0(0%)	1(0.9%)	1(1.9%)	1(0.7%)	0(0%)
**SUBTOTAL**	**340**	**245**	**95**	**112**	**53**	**149**	**26**	**45**	**112**	**53**	**149**	**26**

Almost a third (30.6%) of unpublished trials (104 trials) had closed before completing accrual, most commonly due to poor accrual or sponsor-related issues (such as a change of sponsor priorities or other financial issues). Uncommon reasons for discontinuing accrual included that the PI had left the institution, unacceptable drug toxicity, or lack of drug availability.

## Discussion

Timely publication of clinical research is critical to developing new and effective treatments for cancer and yet, others have previously lamented the under-reporting of oncology trials [5, 6, 11, 18, 19]. Relatively few analyses have been reported on the incidence of publication of clinical oncology trials. Two reports of publication rates of oncology-specific randomized clinical trials found that 66.6% [4] and 62% [20] were ultimately published. However, our study had important differences in criteria for trials inclusion, the methodology used to analyze the rate of publication taking into account censoring and follow-up time, and the definition of the primary endpoints making direct comparisons difficult. Among oncology trials reported as abstracts at the American Society of Clinical Oncology, the percentage of trials eventually published ranged between 61–74% [6, 11, 18, 19] with little difference between phase I, II, or III trials.

Our study reports on 809 oncology trials of all phases from pilot studies to phase III trials from a single cancer center including therapeutic and non-therapeutic trials. We chose to calculate time to publication from the date when patient accrual ended at our center because the date of study closure is often years after completion of accrual and it is common to publish data prior to final closure of the study. Also, from a patient’s point of view, the date of completing accrual represents the completion of patient participation in the clinical research. Starting from that time point, patients (and society) look to the timely publication of results. The estimated probability of publication by 7 years from the date of accrual completion at our center was 70.4% although only 18.8% of trials had been published within 2 years of completing accrual. The median time to publication was 47 months–nearly 4 years. Of unpublished trials, most were either completed or in progress although 45 trials (13.2% of unpublished trials) were completed but not published because the results were not considered of sufficient interest. Up to 2 years after completing accrual, the probability of publication for non-therapeutic trials was the same as for therapeutic trials. After 2 years however, non-therapeutic trials were less likely to be published. In 18.9% of the unpublished non-therapeutic trials, the reason given was that the data were not considered of interest. This is almost twice as frequent as for therapeutic trials (11%) and may speak to a shorter “shelf life” in which non-therapeutic trials that are not published quickly are more likely than therapeutic trials to lose relevance.

Much has been written about outcome reporting bias of industry-sponsored trials but there has been little published regarding the actual likelihood of publication of industry-sponsored trials within oncology. One analysis of randomized oncology trials registered at ClinicalTrial.gov found that industry-sponsored trials were published less often (40%) than nonindustry/nongovernment sponsored trials (56%) [8]. However, another trial evaluating the likelihood of publication among abstracts presented at ASCO found no difference between industry-sponsored and nonindustry-sponsored trials [11]. In our analysis, we saw no overall difference in the probability of publication based on the type of sponsor.

The phase of the trial did not affect the time to publication. Pilot trials and phase I trials were not published any quicker or at higher proportion than phase III trials. We had expected that all pilot and phase I trials would have been published in a relatively short period of time since they are typically smaller trials that do not have survival endpoints. Consistent with this, Table 4 confirms that very few unpublished pilot and phase I trials were still in progress in contrast to 45% of the unpublished phase III trials. Unpublished phase I trials were more likely than the other trials to have been discontinued by the sponsor, and both unpublished pilot and phase I trials were more likely to have been completed but considered not interesting than were the other trials. We have previously reported that lack of time and relocation of authors were common factors affecting the publication of phase I trials [19]. This has also been seen for phase II trials along with lack of interesting results [18] as reasons for failure to publish.

It is often difficult to get manuscripts published reporting negative trial results [1, 2, 5–7]. The scientific community interest level can be low and journals may believe that negative reports adversely affect their impact scores. However, most clinical trial participants believe that they have a right to study result disclosure regardless of trial outcome [21]. Depositing the results on clinicaltrials.gov in a timely manner can make minimum data sets available to the research and patient communities. However, for interventional FDA-regulated drug studies, the FDA Amendments Act only requires reporting of phase II and III trial results [22]. Even for these trials, reporting is not required until a year after all data have been collected. As noted above, oncology trials are often closed to accrual but left open for various reasons such as further survival follow up; full closure is typically several years after completion of accrual. Compounding this problem is the finding that compliance with the requirement to post results on clinicaltrials.gov is not universal. One study found that results had not been reported on clinicaltrials.gov within 4 years of study closure in 29.5% of completed trials [10]. Further, since results of phase I trials are not required to be reported on clinicaltrials.gov, phase I trials that find a new drug treatment inactive or too toxic may not be published anywhere.

One hundred four (30.6%) of the unpublished trials in our analysis were discontinued before completing accrual. Almost half of these cases were due to poor accrual which may indicate a failure to assess resources and patient availability accurately. To minimize the drain on resources and to limit patient involvement in trials that will ultimately fail to accrue, we have implemented institutional mechanisms to identify trials within 18 months that are unlikely to complete accrual and to close them within 30 months if the recruitment issue cannot be corrected. One cause of patient recruitment failure was a result of the PI relocating to another institution in 13 trials (3.8%). Since trial participation by an institution implies sufficient importance of the question being addressed, institutions should establish mechanisms to assure that opened trials will be completed even if the original PI relocates.

This analysis has some shortcomings. The results are limited only to our institution over this 5-year time period. However, we hope these results stimulate other cancer centers to look at their experiences regarding time to publication. Second, for publications indexed to NCT numbers, and unindexed publications that we identified by PI questionnaires, we cannot formally rule out that there could have been an earlier, unindexed publication. Similarly, for the 340 unpublished trials, it is possible that some were published unbeknownst to the PI and despite our NCT number database search. However, we do not think the number of such publications, if any, would have affected our results. A third shortcoming is that, for the trials that were published, we did not study the causes of publication delays. This would be a fruitful exercise for future investigation. For example, it is unclear why the published pilot and phase I trials took as long or longer to publish than phase III trials. We need to consider how long do investigators take to write manuscripts once sufficient data are available? How long does peer review take? How many separate submissions are made before acceptance? How many journals make accepted manuscripts available on line immediately? Finally, where can “negative” data be published so that they are available to the community and the author receives appropriate academic credit for having completed and published the study, which presumably had clinical relevance at the time the trial was opened? How these observations made from oncology trials of all phases relate to the publication barriers for non-oncology clinical trials would seem a valuable area of inquiry, but beyond the scope of our study. We all have a responsibility and an interest in minimizing the time it takes to publish the results of our clinical research. Moreover, our responsibilities to our patients demand it.

## References

[1] Scherer RW, Langenberg P, von Elm E. Full publication of results initially presented in abstracts. The Cochrane database of systematic reviews. 2007;(2):MR000005. doi: 10.1002/14651858.MR000005.pub3 . 17443628 10.1002/14651858.MR000005.pub3

[2] Callaham ML, Wears RL, Weber EJ, Barton C, Young G. Positive-outcome bias and other limitations in the outcome of research abstracts submitted to a scientific meeting. JAMA. 1998;280(3):254–7. . 9676673 10.1001/jama.280.3.254

[3] Emerson GB, Warme WJ, Wolf FM, Heckman JD, Brand RA, Leopold SS. Testing for the presence of positive-outcome bias in peer review: a randomized controlled trial. Archives of internal medicine. 2010;170(21):1934–9. doi: 10.1001/archinternmed.2010.406 . 21098355 10.1001/archinternmed.2010.406

[4] Chen YP, Liu X, Lv JW, Li WF, Zhang Y, Guo Y, et al Publication status of contemporary oncology randomised controlled trials worldwide. Eur J Cancer. 2016;66:17–25. doi: 10.1016/j.ejca.2016.06.010 . 27522246 10.1016/j.ejca.2016.06.010

[5] De Bellefeuille C, Morrison CA, Tannock IF. The fate of abstracts submitted to a cancer meeting: factors which influence presentation and subsequent publication. Ann Oncol. 1992;3(3):187–91. . 1586615 10.1093/oxfordjournals.annonc.a058147

[6] Krzyzanowska MK, Pintilie M, Tannock IF. Factors associated with failure to publish large randomized trials presented at an oncology meeting. JAMA. 2003;290(4):495–501. doi: 10.1001/jama.290.4.495 . 12876092 10.1001/jama.290.4.495

[7] Johnson RT, Dickersin K. Publication bias against negative results from clinical trials: three of the seven deadly sins. Nat Clin Pract Neuro. 2007;3(11):590–1. 10.1038/ncpneuro0618 17876349

[8] Ross JS, Mulvey GK, Hines EM, Nissen SE, Krumholz HM. Trial Publication after Registration in ClinicalTrials.Gov: A Cross-Sectional Analysis. PLoS Med. 2009;6(9):e1000144 doi: 10.1371/journal.pmed.1000144 19901971 10.1371/journal.pmed.1000144 PMC2728480

[9] Ross JS, Mocanu M, Lampropulos JF, Tse T, Krumholz HM. TIme to publication among completed clinical trials. JAMA Internal Medicine. 2013;173(9):825–8. doi: 10.1001/jamainternmed.2013.136 23460252 10.1001/jamainternmed.2013.136 PMC3691813

[10] Saito H, Gill CJ. How frequently do the results from completed US clinical trials enter the public domain?—A statistical analysis of the ClinicalTrials.gov database. PLoS One. 2014;9(7):e101826 doi: 10.1371/journal.pone.0101826 ; PubMed Central PMCID: PMC4098992. 25025477 10.1371/journal.pone.0101826 PMC4098992

[11] Massey PR, Wang R, Prasad V, Bates SE, Fojo T. Assessing the Eventual Publication of Clinical Trial Abstracts Submitted to a Large Annual Oncology Meeting. Oncologist. 2016;21(3):261–8. doi: 10.1634/theoncologist.2015-0516 ; PubMed Central PMCID: PMC4786361. 26888691 10.1634/theoncologist.2015-0516 PMC4786361

[12] Smithy JW, Downing NS, Ross JS. Publication of pivotal efficacy trials for novel therapeutic agents approved between 2005 and 2011: A cross-sectional study. JAMA Internal Medicine. 2014;174(9):1518–20. doi: 10.1001/jamainternmed.2014.3438 25070357 10.1001/jamainternmed.2014.3438 PMC4221247

[13] Chen R, Desai NR, Ross JS, Zhang W, Chau KH, Wayda B, et al Publication and reporting of clinical trial results: cross sectional analysis across academic medical centers. Bmj. 2016;352:i637 doi: 10.1136/bmj.i637 ; PubMed Central PMCID: PMC4768882. 26888209 10.1136/bmj.i637 PMC4768882

[14] Ross JS, Tse T, Zarin DA, Xu H, Zhou L, Krumholz HM. Publication of NIH funded trials registered in ClinicalTrials.gov: cross sectional analysis. Bmj. 2012;344:d7292 doi: 10.1136/bmj.d7292 ; PubMed Central PMCID: PMC3623605. 22214755 10.1136/bmj.d7292 PMC3623605

[15] Kasenda B, von Elm E, You J, et al Prevalence, characteristics, and publication of discontinued randomized trials. JAMA. 2014;311(10):1045–52. doi: 10.1001/jama.2014.1361 24618966 10.1001/jama.2014.1361

[16] Iasonos A, Chapman PB, Satagopan JM. Quantifying Treatment Benefit in Molecular Subgroups to Assess a Predictive Biomarker. ClinCancer Res. 2016;22(9):2114–20. doi: 10.1158/1078-0432.ccr-15-2517 27141007 10.1158/1078-0432.CCR-15-2517 PMC4856220

[17] Davis FG, McCarthy BJ, Freels S, Kupelian V, Bondy ML. The conditional probability of survival of patients with primary malignant brain tumors: surveillance, epidemiology, and end results (SEER) data. Cancer. 1999;85(2):485–91. . 10023719

[18] Hoeg RT, Lee JA, Mathiason MA, Rokkones K, Serck SL, Crampton KL, et al Publication outcomes of phase II oncology clinical trials. Am J Clin Oncol. 2009;32(3):253–7. doi: 10.1097/COC.0b013e3181845544 . 19349853 10.1097/COC.0b013e3181845544

[19] Camacho LH, Bacik J, Cheung A, Spriggs DR. Presentation and subsequent publication rates of phase I oncology clinical trials. Cancer. 2005;104(7):1497–504. doi: 10.1002/cncr.21337 . 16116590 10.1002/cncr.21337

[20] Schandelmaier S, Conen K, von Elm E, You JJ, Blumle A, Tomonaga Y, et al Planning and reporting of quality-of-life outcomes in cancer trials. Ann Oncol. 2015;26(9):1966–73. doi: 10.1093/annonc/mdv283 ; PubMed Central PMCID: PMCPMC4551161. 26133966 10.1093/annonc/mdv283 PMC4551161

[21] Elzinga KE, Khan OF, Tang AR, Fernandez CV, Elzinga CL, Heng DY, et al Adult patient perspectives on clinical trial result reporting: A survey of cancer patients. Clinical Trials. 2016;13(6):574–81. doi: 10.1177/1740774516665597 27559022 10.1177/1740774516665597

[22] Zarin DA, Tse T, Williams RJ, Carr S. Trial Reporting in ClinicalTrials.gov—The Final Rule. NEnglJMed. 2016;375(20):1998–2004. doi: 10.1056/NEJMsr1611785 . 27635471 10.1056/NEJMsr1611785 PMC5225905

